# Association between copy-number alteration of +20q, −14q and −18p and cross-sensitivity to tyrosine kinase inhibitors in clear-cell renal cell carcinoma

**DOI:** 10.1186/s12935-020-01585-1

**Published:** 2020-10-06

**Authors:** Liang Wang, Yuqing Li, Yinfeng Lyu, Hui Wen, Chenchen Feng

**Affiliations:** 1 Department of Urology, Tianjin Medical University General Hospital, Tianjin, 300052 People’s Republic of China; 2 Department of Urology, Huashan Hospital, Fudan University, Shanghai, 200040 People’s Republic of China

**Keywords:** Clear-cell renal cell carcinoma, Copy-number alteration, Tyrosine kinase inhibitor, Cross-sensitivity

## Abstract

**Background:**

We aim to explore association between copy number alteration (CNA) and sensitivity to common tyrosine kinase inhibitors (TKIs) used in clear-cell renal cell carcinoma (ccRCC) treatment.

**Methods:**

CNA with related sensitivity profiles were extracted from the Genomics of Drug Sensitivity in Cancer (GDSC) dataset and was cross-referenced with common CNA in ccRCC in the Cancer Genome Atlas (TCGA) dataset. Functional annotation was profiled using GSEA and NET-GE. Target genes within cytobands of interest were screened in silico and validated in vitro using proliferation assays in A498 and 786-O ccRCC cells.

**Results:**

Four TKIs (Sunitinib, Cabozantinib, Axitinib and Sorafenib) that were clinically used in ccRCC were selected. In silico analysis showed gain of 20q (+20q) occurred in ~ 23% of cases and was associated with resistance to all four TKIs; loss of 14q (−14q) occurred in ~ 39% of cases and was associated with resistance to Sunitinib and Sorafenib; loss of 18p (−18p) occurred in ~ 39% of cases and was associated with sensitivity to Sunitinib and Sorafenib. All 3 CNAs were associated with worsened prognosis, respectively. Candidate target genes included of RBL1 on 20q, KLHL33 on 14q and ARHGAP28 on18q. In vitro validation showed RBL1 overexpression induced resistance to Sunitinib and Cabozantinib; KLHL33 silencing induced resistance to Sunitinib; ARHGAP28 silencing induced sensitivity to Cabozantinib. Functional annotation indicated FoxO signaling, hypoxic response and Wnt pathway, and Rho-related cellular adhesion were mechanistically associated with +20q, −14q and −18p, respectively.

**Conclusion:**

Common CNAs in ccRCC are associated with cancer-intrinsic cross-sensitivity to common TKIs. Further validation and functional analyses are therefore needed.

## Background

Clear-cell renal cell carcinoma (ccRCC) is the most common type of RCC and poses a major health threat worldwide [1]. Whilst ~ 70% of cases are localized or locally advanced that can be treated with extirpative surgery with or without adjuvant tyrosine kinase inhibitors (TKIs) that entails prolonged survival [2–4], ~ 30% of cases are diagnosed with initial metastatic disease with a median overall survival of 43.2 months, 22.5 months and 7.8 months corresponding to favorable to poor risk groups by International Metastatic RCC Database (IMDC) criteria [5]. TKIs target tyrosine kinases which are enzymes responsible for the activation of a variety of proteins by signal transduction cascades [6]. The proteins are activated by phosphorylation, a step that TKIs inhibit [6]. Most TKIs are typically used as anticancer drugs acting both in cancer-intrinsic and/or micro-environmental setting [7]. The IMDC risk group category reflects comprehensive survival data worldwide in the era of TKIs for metastatic renal cell carcinoma (mRCC) and in spite of newly emerging immune checkpoint inhibitors (ICIs) taking up frontlines of mRCC treatment, TKIs still play critical roles as TKI + ICI combo now stands at the standard of care for mRCC as revealed by latest randomized trials [8–11].

Copy number alteration (CNA) plays critical role in carcinogenesis. Unlike point mutations that requires persistent selection stress, DNA rearrangements can dynamically alter more swiftly throughout cancer evolution. Thus far, the 2 massive deep sequencing studies, namely the Cancer Genome Atlas (TCGA) and TRACERx (TRAcking Cancer Evolution through therapy (Rx)) projects have revealed instantaneous and evolutionary alteration of CNA in ccRCC, showing truncal events of 3p loss and 5q gain [12–15]. Other CNAs that occur at a lesser frequency also play critical roles as the genotypes often represent distinct phenotypes. Chromosome 14q loss is reported to define a molecular subtype of clear-cell renal cell carcinoma associated with poor prognosis [16]. Deletion of 18q has also been reported to confer worsened prognosis [17]. Likewise, 9p loss has been validated in multiple studies to be associated with poor outcome [18–21]. However, there are only a handful of studies that link CNAs with treatment response in ccRCC.

In the current study, we have investigated associations between sensitivity to commonly used tyrosine kinase inhibitors (TKIs) and CNAs in ccRCC using an in silico exploration with in vitro validation.

## Materials and methods

### In silico analysis

#### Reproduction of genomics of drug sensitivity in cancer (GDSC) dataset

The GDSC dataset contained CNA profile of cancer cells labeled as cnaPANCAN with each number corresponding to a specific cytoband [22]. We started by querying 4 clinically used TKIs in ccRCC, namely Sunitinib, Cabozantinib, Axitinib and Sorafenib with the last having 2 datasets (GDSC1 and GDSC2). We then selected genomic cnaPANCAN profiles significantly associated with sensitivity or resistance to each of the TKIs with P value of < .05. We did not designate q value as we consider the pan-cancer cell lines were solely studied as an exploration. Candidate CAN cytobands were cross-referenced between each TKI profiles and only overlapping cnaPANCAN labels were allowed for further validation in ccRCC.

#### Reproduction of The Cancer Genome Atlas (TCGA) dataset

Reproduction of TCGA dataset was performed on the cBioPortal online platform (http://www.cbioportal.org/) with the selection of KIRC (Kidney clear cell carcinoma) Firehose Legacy subsets [23, 24]. Copy number variance (CNV) dataset was used with GISTIC value of > 2 designated as amplification and between 1 and 2 designated as gain. Target genes of interest including RBL1, KLHL33 and ARHGAP28 was queried in KIRC dataset. Gene enrichment analysis for CNA cases was first analyzed using the NETwork-based Gene Enrichment (http://net-ge.biocomp.unibo.it/enrich) and then validated using the GSEA approach [25] with mRNA expression data (RNA seq V2) retrieved from TCGA. Each cytoband was queried using several benchmark genes within the chromosome (Chr). For +20q we used AAR2, ADA and ADRM1; for −14q we used HIF1A, LRP10 and APEX; for −18q we used ADCYAP1. Correlations of mRNA expression against corresponding copy number value were also analyzed and plotted with cBioPortal. Survival analysis for CNA was analyzed using cBioPortal and for mRNA expression was performed at Human Protein Atlas platform (https://www.proteinatlas.org/). Expression cutoff for expression of genes (high vs. low) was automatically designated by the platform in the survival analysis.

#### Reproduction of Gene Expression Omnibus (GEO) repository

Two GEO accessions (GSE64052 and GSE66346) were used to validate expression of gene of interest and sensitivity to Sunitinib [26, 27]. The expression data of cohorts of interest was retrieved online (https://www.ncbi.nlm.nih.gov/geo/) and analyzed with the Student’s *t* test using the Prism Graphpad ver 7 software.

### In vitro assays

#### Cell lines and target gene selection

Both 786-O and A498 clear-cell renal cell carcinoma cells were purchased from the cell bank of Chinese Academy of Science and cultured in RPMI-1640 medium. We selected of potential target gene on 20q, 14q and 18p by first pinpointing significantly enriched pathways. Then we selected genes within both the pathways and located on the chromosome. At last, we used RNAi to test proliferation of cells. Negative genes were omitted in the current study and potential target genes included RBL1 on 20q, KLHL33 on 14q and ARHGAP28 on 18p. We used TRC to construct shRNA targeting KLHL33 and ARHGAP28 (TRC, http://www.broadinstitute.org/rnai/public/). Two transcripts were selected for each gene (TRCN0000253673 and TRCN0000253672 for KLHL33; TRCN0000144667 and TRCN0000142196 for ARHGAP28). cDNA clones for overexpression of RBL1 was purchased from Origene (RC207017L3V). Vectors with resistance to puromycin were constructed and transfected via non-lipofectamine fugene transfection. After incubation, positive clones were selected by puromycin supplement and control vectors were generated with similar approach.

#### Proliferation assay

Proliferation was studied using the crystal violet assay as previously reported by our group [28, 29]. Briefly, stably transfected cells seeded in 96-well plate were treated with Sunitinib, Cabozantinib, Axitinib and Sorafenib at indicated doses (Additional file 1: Figure S1), respectively. Treatment was given twice every 48 h with change of media. At 96 h, supernatant was discarded and plates were rinsed followed by fixation with formalin. Cells were then stained using crystal violet and dissolved using methanol. Plates were processed on a plate reader with absorbance at 540 nm. Data were presented as relative percentage to control group at 0 h.

### Statistical analyses

Prism Graphpad ver.7 for Mac was used for graph generation and statistical analysis. Data were presented as mean ± standard deviation (SD). The means were compared using the Student’s *t* test and medians were compared using the Mann–Whitney’s U-test. The Kaplan–Meier curve was used for survival data which was compared using log-rank test. The P value of < .05 was accepted as statistically significant.

## Results

### Cross–sensitivity or—resistance and CNAs in ccRCC

**Fig. 1:**
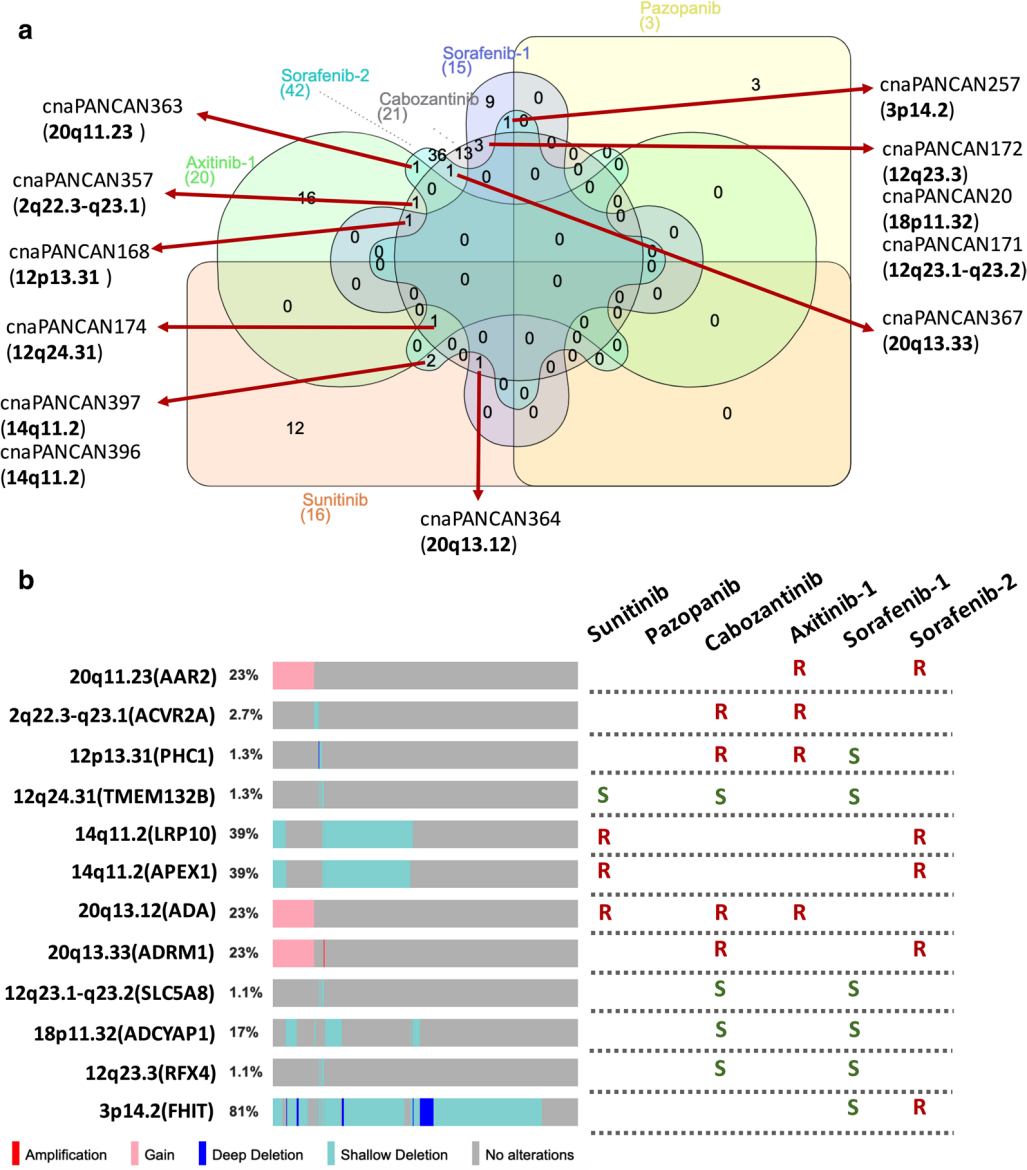
Copy-number alterations (CNAs) and cross-resistance and –sensitivity to tyrosine kinase inhibitors (TKIs). **a** Reproduced from Genomics of Drug Sensitivity in Cancer (GDSC) dataset, shown was Venn diagram of each CNAs (GDSC labels and corresponding cytobands) with significant sensitivity or resistance to TKIs in pan-cancer cells; **b** Reproduced from the Cancer Genome Atlas (TCGA) renal clear cell carcinoma (KIRC) dataset, shown was frequency of CNAs with queried genes in brackets and sensitivity profile in GDSC dataset

To comprehensively investigate CNAs and sensitivity to TKIs, we first queried 5 sub-datasets (2 sets for Sorafenib) in GDSC encompassing 4 clinically used drugs using the pan-cancer cell line data. Surprisingly, we found that cross -sensitivity or –resistance to those agents were common (Fig. 1a). We then plotted CNA of each cytoband to examine the frequency in ccRCC and found high frequency of −3p, +20q, −14q and −18p (Fig. 1b). We then tried to determine which CNAs were of investigational merit and thus designated that only CNAs with frequency over 10% and with consistent sensitivity profile were selected and −3p was excluded. Of note, cross–sensitivity or –resistance was common amongst those TKIs. +20q showed cross resistance to all four TKIs, whilst −14q and −18q each show cross –resistance and –sensitivity to 2 drugs, respectively (Fig. 1b).

### +20q and resistance to Sunitnib and Cabozantinib

**Fig. 2:**
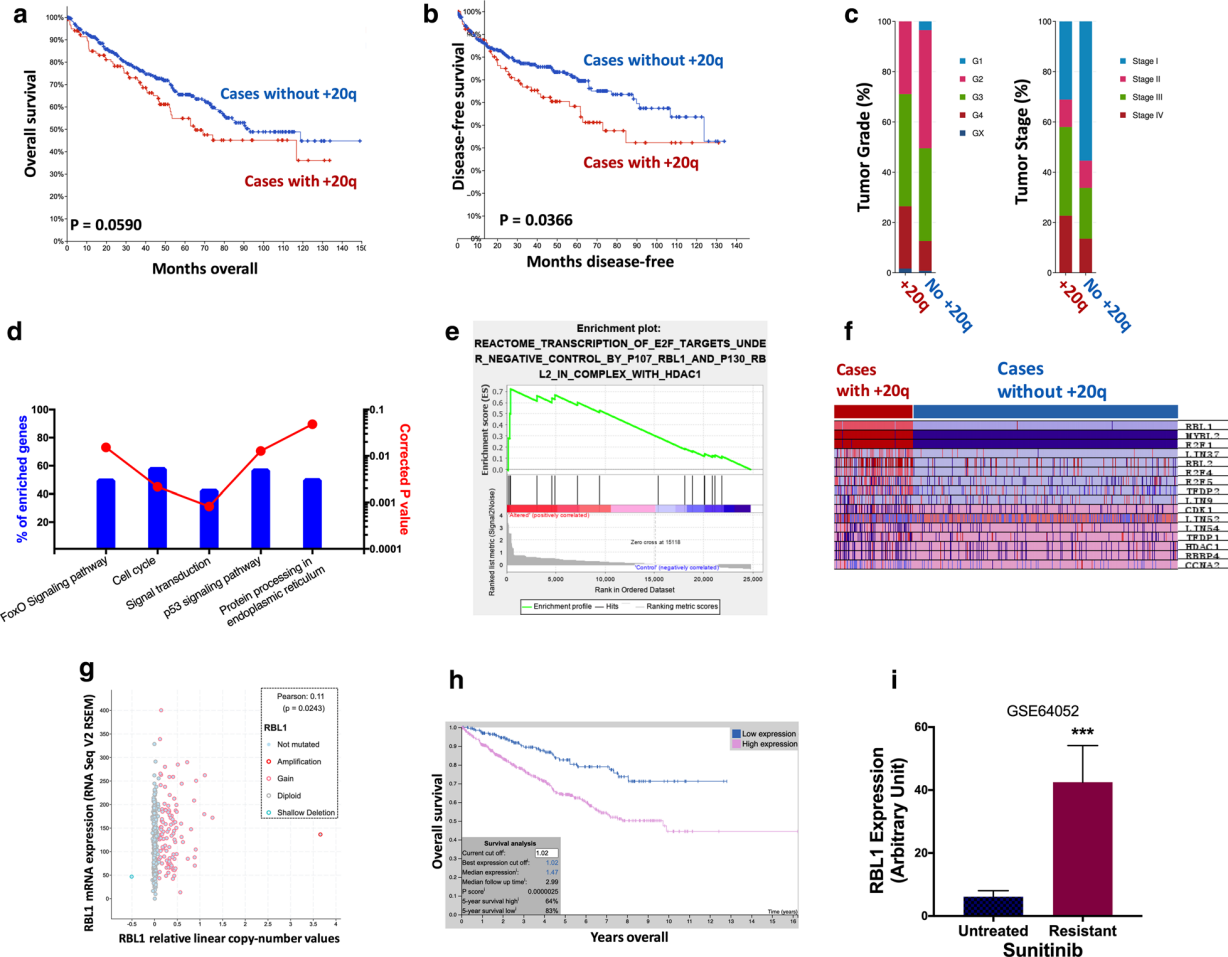
+20q and resistance to Sunitinib and Cabozantinib. Reproduced from TCGA, shown were **a** overall survival, **b** progression-free survival and **c** clinicopathological parameters of patient with and without +20q; Gene enrichment and functional annotation analysis in TCGA cases with or without +20q using the **d** NET-GE and **e** GSEA platforms with **f** genes of interest shown in heatmap; **g** Reproduced from TCGA dataset, shown was correlation between copy number against corresponding mRNA expression of the gene of interest; **h** Reproduced from TCGA and Human Protein Atlas dataset, shown was overall survival of patients with higher and lower RBL1 expression with automatically designated cutoff value; **i** Reproduced from GEO dataset, shown was RBL1 expression in Sunitinib –untreated and –resistant RCC cells (Data were presented as mean ± standard deviation; *P < 0.05; **P < 0.01; ***P < 0.001)

+20q has already been reported in ccRCC yet the target gene thereon as well as biological significance remained elusive [30]. Here we showed ccRCC cases with +20q had worsened overall prognosis (Fig. 2a) with marginal significance (P = 0.059). Such cases also had significantly worsened progression-free survival (P = 0.0366) (Fig. 2b). +20q cases had significantly more high-grade (P = 7.920e-5) and late-staged (P = 2.086e-5) tumors compared to its counterpart (Fig. 2c). NET-GE-based pathway analysis revealed genes within FoxO (P = 1.528e-02), cell cycle (P = 2.152e-03) and p53 (P = 1.280e-02) signaling significantly enriched (Fig. 2d). In the parallel GSEA analysis, genes within FoxO signaling was also enriched in +20q cases (Fig. 2e). We then pinpointed the FoxO-related gene RBL1 located on 20q showing strong differential expressions between two cohorts (Fig. 2f). RBL1 showed a significant positive correlation (P = 0.0243) between CNA and mRNA expression indicating a functional gene product (Fig. 2g). Higher expression of RBL1 was associated with significantly worsened prognosis in renal cell carcinoma (P = 2.5e-06) (Fig. 2h). External validation using GEO dataset showed significantly higher RBL1 expression in Sunitinib-resistant ccRCC cells (P < 0.001) (Fig. 2i). In the proliferation assays, we examined effect of RBL1 overexpression at different doses and found that only resistance to Sunitnib and Cabozantinib was observed whilst proliferation was not altered in ccRCC cell treated with Axitinib or Sorafenib (Additional file 1: Figure S1).

### -14q and resistance to Sunitinib

**Fig. 3:**
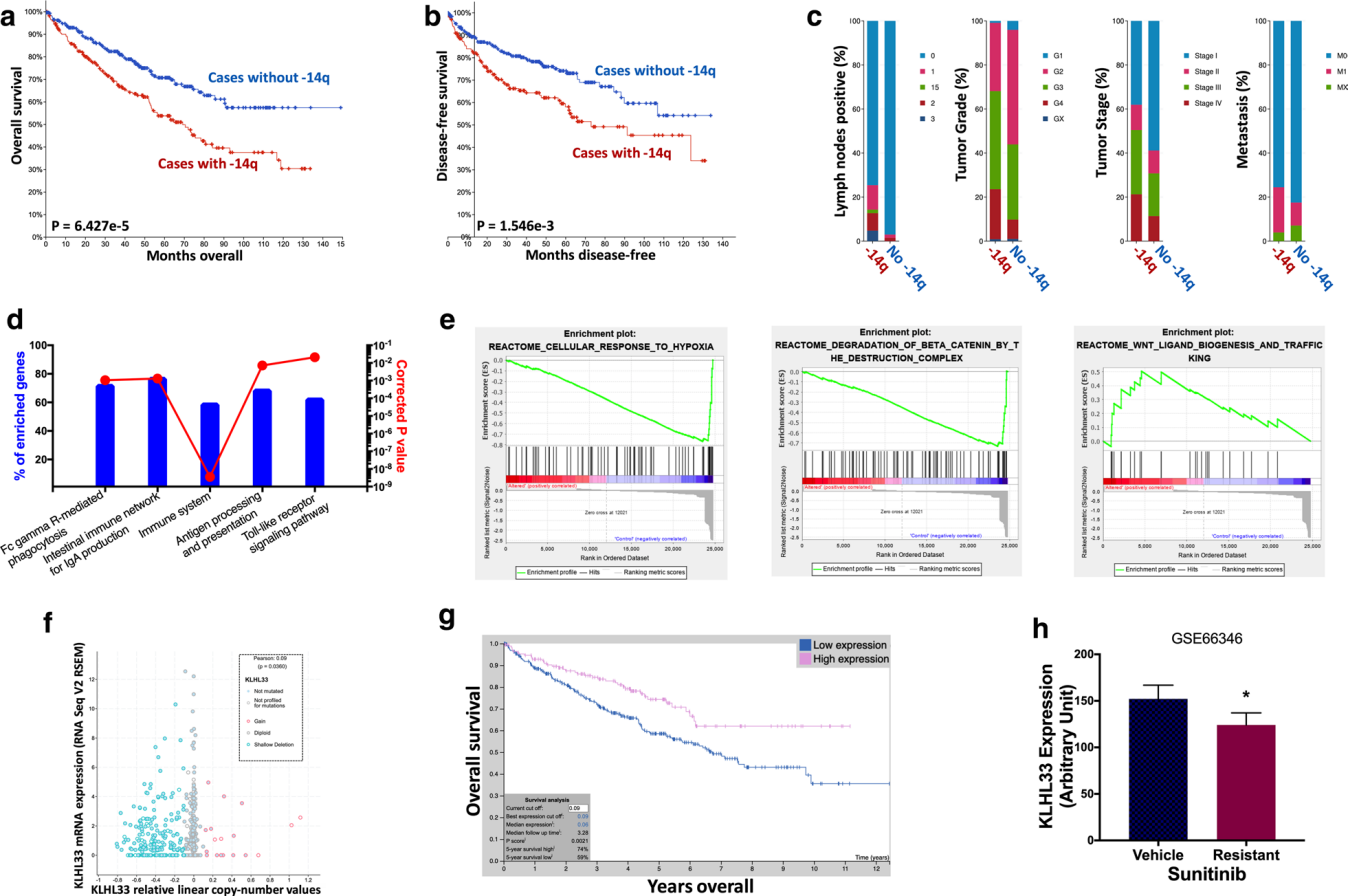
−14q and resistance to Sunitinib. Reproduced from TCGA, shown were **a** overall survival, **b** progression-free survival and **c** clinicopathological parameters of patient with and without −14q; Gene enrichment and functional annotation analysis in TCGA cases with or without −14q using the **d** NET-GE and **e** GSEA platforms; **f** Reproduced from TCGA dataset, shown was correlation between copy number against corresponding mRNA expression of the gene of interest; **g** Reproduced from TCGA and Human Protein Atlas dataset, shown was overall survival of patients with higher and lower KLHL33 expression with automatically designated cutoff value; **h** Reproduced from GEO dataset, shown was KLHL33 expression in Sunitinib –untreated and –resistant RCC cells (Data were presented as mean ± standard deviation; *P < 0.05)

-14q was also reported in ccRCC with one of its target gene HIF1A of interest. Here we showed ccRCC cases with -14q had worsened overall prognosis (P = 6.427e-5) (Fig. 3a) and progression-free survival (P = 1.546e-3) (Fig. 3b). −14q cases had significantly more nodal positive tumors (P = 7.483e-4), high-grade (P = 7.99e-8) tumors, late-staged tumors (P = 1.885e-5) and metastatic tumors (P = 2.617e-3) compared to its counterpart (Fig. 3c). Interestingly, NET-GE-based pathway analysis showed significant enrichment of genes within immune-related pathways (Fig. 3d). GSEA analysis however showed no significant enriched pathways in −14q cases yet cases without −14q showed significant enrichment in hypoxia and canonical Wnt signaling (P < 0.0001) (Fig. 3e). As both immunity and hypoxia requires microenvironment interaction, we aimed at genes within Wnt signaling that was cancer-intrinsic. We pinpointed KLHL33 that was located on 14q belonging to the KLHL family which played important role in Wnt mediation. KLHL33 showed a significant positive correlation between CNA and mRNA expression indicating a functional gene product (P = 0.036) (Fig. 3f). Lower expression of KLHL33 was associated with significantly worsened prognosis in renal cell carcinoma (P = 0.0021) (Fig. 3g). External validation using GEO dataset showed significantly lower KLHL33 expression in Sunitinib-resistant ccRCC cells (P < 0.05) (Fig. 3h). In the proliferation assays, we examined effect of KLHL33 silencing at different doses and found that only resistance to Sunitnib was observed whilst proliferation was not altered in ccRCC cell treated with Sorafenib (Additional file 1: Figure S1).

### −18q and sensitivity to Cabozantinib and Sorafenib

**Fig. 4:**
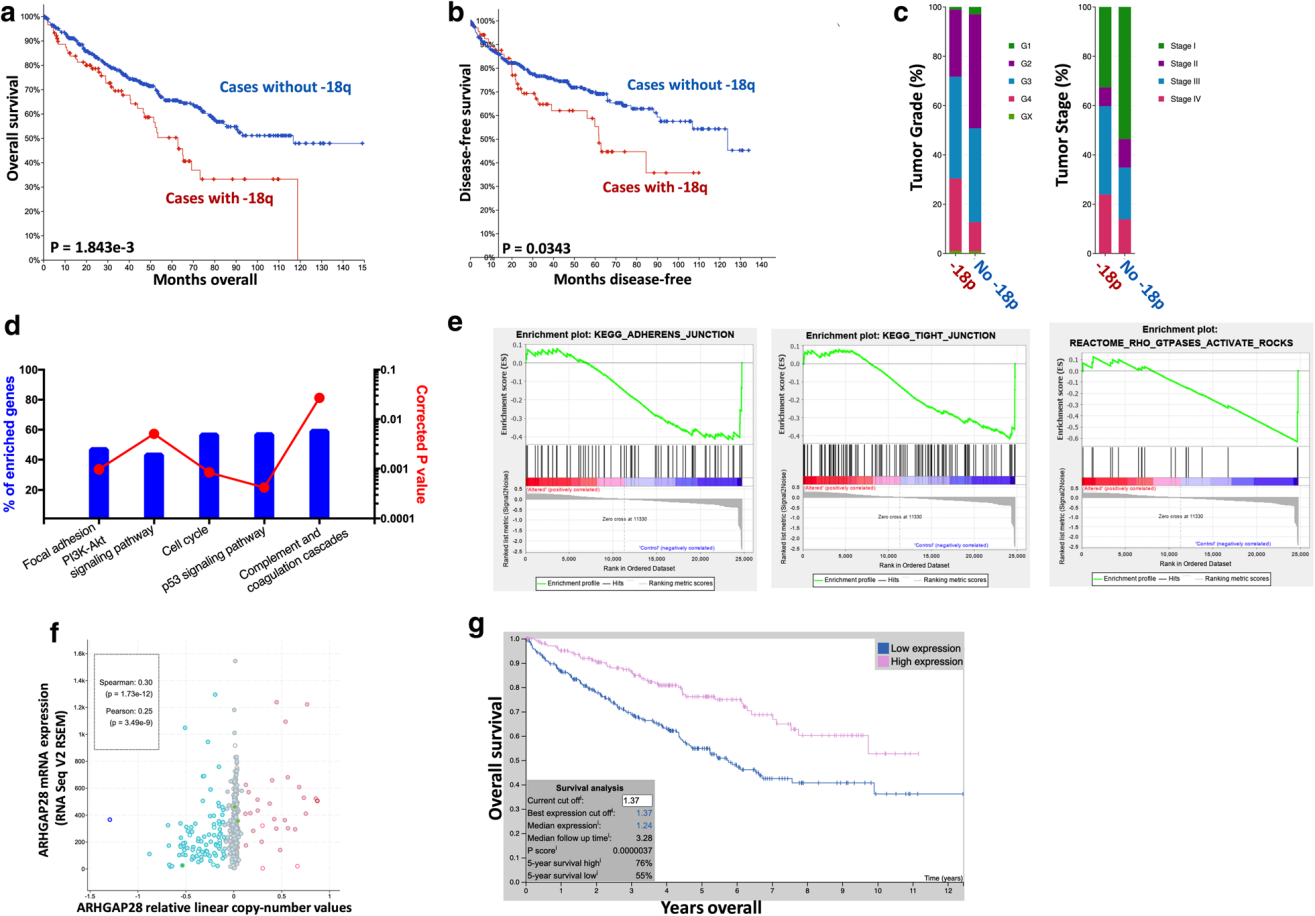
−18q and sensitivity to Cabozantinib and Sorafenib. Reproduced from TCGA, shown were **a** overall survival, **b** progression-free survival and **c** clinicopathological parameters of patient with and without +20q; Gene enrichment and functional annotation analysis in TCGA cases with or without +20q using the **d** NET-GE and **e** GSEA platforms with genes of interest shown in heatmap; **f** Reproduced from TCGA dataset, shown was correlation between copy number against corresponding mRNA expression of the gene of interest; **g** Reproduced from TCGA and Human Protein Atlas dataset, shown was overall survival of patients with higher and lower ARHGAP28 expression with automatically designated cutoff value

−18q has also been reported in ccRCC with indication of more aggressive phenotype [31]. Here we showed ccRCC cases with -20q had worsened overall prognosis (P = 1.843e-3) (Fig. 4a) and progression-free survival (P = 0.0343) (Fig. 4b). −18q + cases had significantly more high-grade (P = 1.037e-4) and late-staged (P = 1.893e-4) tumors compared to its counterpart (Fig. 4c). NET-GE-based pathway analysis revealed gene within focal adhesion (P < 0.001), PI3K/Akt signaling (P < 0.001) and cell cycle regulation (P = 4.233e-04) significantly enriched (Fig. 4d). In GSEA analysis, genes within Adherens, tight junction and Rho signaling was also enriched in −18q cases (Fig. 4e). We then pinpointed the Rho-related gene ARHGAP28, which was located on 14q showing significant positive correlation between CNA and mRNA expression indicating a functional gene product (P = 1.73e-12) (Fig. 4f). Lower expression of ARHGAP28 was associated with significantly worsened prognosis in renal cell carcinoma (P = 3.7e-6) (Fig. 4g). In the proliferation assays, we examined effect of ARHGAP28 silencing at different doses and found that sensitivity to Cabozantinib and Sorafenib (Additional file 1: Figure S1).

## Discussion

In the current study, we identified several CNAs that were associated with cross sensitivity to TKIs in ccRCC. Although TKIs in ccRCC mainly target angiogenesis which was a major feature of the cancer, those multi-target agents also provided functional blockade to cancer-intrinsic pro-tumorigenic pathways. Those targets could overlap between TKIs and cross-resistance was common [32]. In the current study, we sought to analyze association between CNAs and TKI sensitivity and we surprisingly found that cross-sensitivity associated with common CNAs in ccRCC. As we aimed only at minor CNAs besides 3p and 5q in our study, those CNAs could very likely be clonal due to treatment stress.

We first found +20q featuring overexpression of RBL1 and enriched FoxO signaling is associated with resistance to both Sunitinib and Cabozantinib, despite that in silico exploration shows resistance to all 4 TKIs. Common cancer-intrinsic targets between Sunitinib and Cabozantinib converge to Akt/PI3K signaling which has extensive crosstalk with FoxO signaling. We speculate that RBL1 participates in cell cycle regulation downstream of FoxO signaling and thus by pass the inhibition by Sunitinib and Cabozantinib, both of which were shown to mediate cell cycle arrest as the cancer-intrinsic mechanism [33, 34]. On chromosomal level, there was a report that Chr20q amplification accounted for only 3.8% of 79 ccRCC cases who received whole genome profiling of chromosomal aberrations [35], while another research identified amplification on 20q with a proportion of 20% based on 90 patients [36]. Beroukhim et al. [36] implemented statistical method GISTIC to identify potential copy number alterations as driver events of tumorigenesis, and screened out several peak regions of gain or loss. They suggested 20q be regarded as tumor suppressor gene (TSG) target due to more than 20 significantly overexpressed genes located on this chromosome arm. These genes include RGS19, TPD52L2, TNFRSF6B, C20orf11 PRPF6, BIRC7, RTEL1 and SOX18. However, it is probable that TSG targets of these expression-altered genes or copy-number changes were replaced by passenger events occurring nearby them. Moore et al. indicated that 25.5% of 412 ccRCC cases are detected 20q gain, which is significantly associated with stage rather than grade. Particularly, both grade and stage are associated with gain of 20q13, a region harboring TP53NK and SALL4 genes. Besides, Chr20 gain appears frequently in late-stage tumors and is observed only in high-grade disease. Interestingly, males more often harbor 20q gain [37, 38].

Cross-resistance to Sunitinib and Sorafenib was also indicated in −14q yet in vitro assays only validated Sunintinb. Based on our findings that hypoxia pathway was enriched in only cases without −14q, we speculate that established target gene HIF1A on 14q plays a critical role upon its presence or deletion. We found that −14q cases show enriched immune modulation pathway, indicating that ccRCC without HIF1A may be more sensitive to ICI rather than angiogenesis-targeting TKIs. On the other hand, we found Wnt pathway genes were marginally enriched in -14q cases. Wnt signaling has been reported to play a role in Sunitinib resistance [39, 40]. We primarily identified KLHL33 as potential target gene. Nonetheless, KLHL33 silencing was not associated with resistance to Sorafenib. We therefore consider −14q a relatively distinct genotype. This corresponds to previous reports. Basically, 14q has been considered a key attribution to poor clinical outcome and recurrence after surgery. Apart from the loss of 3p in 99% of samples, it was detected that loss of 14q accounted for 55% of samples and ranked the second most frequent copy number variations. It was also found that 14q loss is associated with significantly decreased overall survival and may be used as one of recurrence biomarkers for organ confined tumors [16]. Notably, the evidence showed the association between 14q loss and diminished mRNA and protein expression of HIF1α, which is located on chromosome 14q23.2 and was often identified lost in high-grade ccRCC [41]. Meanwhile, the stabilization of hypoxia inducible factor(HIF) is also influenced by the trunk event 3p loss and VHL gene inactivation in ccRCC. Moore [38] et al. took array comparative genomic hybridization to recognize copy number alterations in ccRCC and found 14q loss took place in 46.8% of patients and had a strong association with tumor grade (p < 0.0001) and a lower extent with stage (p = 0.006). Furthermore, this type of chromosome aberration was found to be correlated with progression and metastases of disease for clear-cell RCC and may define a subtype of RCC [42].

Interestingly, we found that loss of ARHGAP28 located on 18p is associated with cancer-intrinsic sensitivity to Cabozantinib and Sorafenib. ARHGAP28 plays an important role in Rho-mediated cell adhesion establishment. Whereas Sorafenib has been shown to mediate adhesion, there is so far no reports on Cabozantinib [43]. However, given the cancer-intrinsic targets of Cabozantinib, both MET and AXL have been shown to participate in cell adhesion [44, 45]. Unlike 3p loss and 5q gain, deep loss of 18p is much less frequent in clear cell renal cell carcinoma. A large study [38] used array comparative genomic hybridization to identify clinicopathological characteristics attributed to CNA in ccRCC and reported that loss of 18p11.2-12 was associated with both tumor grade and stage. Similarly, another study [35] of 80 cases showed evidence for close correlation between 18p loss and tumor stage (p = 0.038). In addition, the telomere lengths of tumor cells were measured and came to a conclusion that tumor samples with loss of heterozygosity (LOH) at chromosome 14q and 18p had shorter telomeres statistically to the borderline significance. Then we could hypothesize that 18p loss imply the malignancy of tumors. Indeed, Chr8 loss rarely occurred in early stage tumors and was observed appeared as tumor proceed and advance in stage, finally peaking at the last stage. This pattern may be caused by genomic material instability along with process of tumor, which denies the hypothesis that loss of Chr8 may act as driver event for tumor proliferation, invasion or metastasis. Furthermore, Chr18 loss was reported detective only when tumor developed into high-grade [37]. Nevertheless, we can’t exclude the possibility that 18p loss is involved in tumorigenesis and progression.

Our study has limitations. First, the in vitro validation in our study is still premature. Substantially more functional analysis should be performed to consolidate the findings. Second, due to lack of ccRCC cell lines with specific CNAs queried in our study, we used RNA interference of potential target gene to simulate effect of chromosomal re-arrangement. Further studies include tissue sample studies that can better validate CNA and treatment response, and biological assays in patient-derived cells with CNA of interest.

In all, we postulate that +20q mediates cross-resistance via FoxO signaling. We also found that −14q was associated with resistance to Sunitinb and the enriched immune modulation could imply an increased response to ICI. Both alterations together with the frequency of CNAs in part elucidate the initial response rate of ccRCC patients to upfront TKIs. Finally, we reported that −18p could be sensitive to Cabozantinib via Rho-mediated cell adhesion regulation. All those findings warrant further mechanistic validation which is now under investigation.

## Supplementary information

**Additional file 1: Figure S1.** In vitro validation using crystal violet proliferation assays in 786-O and A498 clear-cell renal cell carcinoma cells. Cells were stably transfected with virus bearing corresponding gene cDNA or shRNA. Proliferations were examined at 96 h of treatment in all assays. (Data were presented as mean ± standard deviation; *P < 0.05; **P < 0.01)

## Data Availability

Not applicable.
