# Supplementary figures and images for: Association between copy-number alteration of +20q, −14q and −18p and cross-sensitivity to tyrosine kinase inhibitors in clear-cell renal cell carcinoma
Source: Cancer Cell Int. 2020 Oct 6;20:482. doi: 10.1186/s12935-020-01585-1 (PMC7541266; doi:10.1186/s12935-020-01585-1)

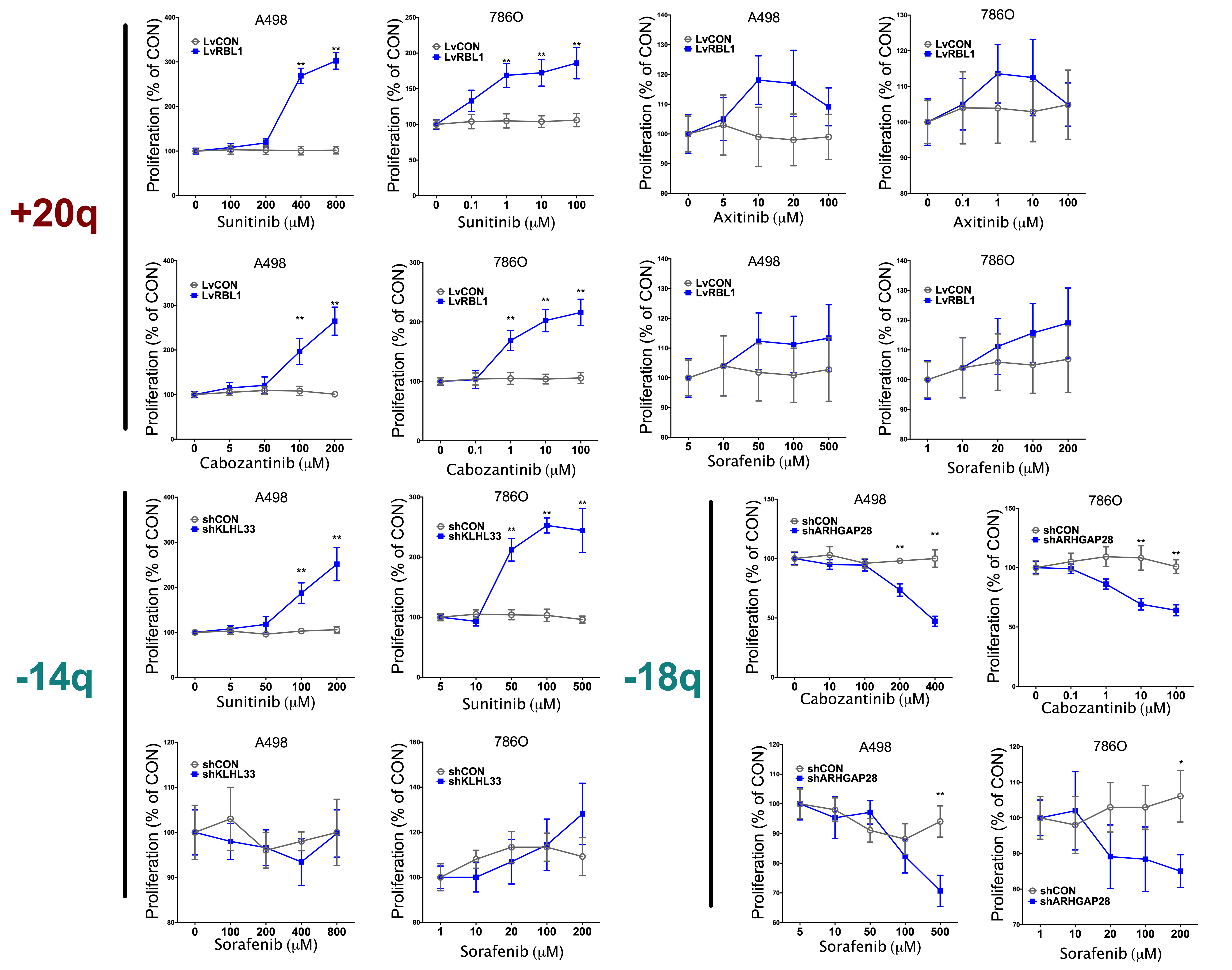

Supplement: Supplementary file 1 — Additional file 1: Figure S1. In vitro validation using crystal violet proliferation assays in 786-O and A498 clear-cell renal cell carcinoma cells. Cells were stably transfected with virus bearing corresponding gene cDNA or shRNA. Proliferations were examined at 96 h of treatment in all assays. (Data were presented as mean ± standard deviation; *P < 0.05; **P < 0.01) [file 12935_2020_1585_MOESM1_ESM.tiff]
